# Pathogenic group 2 innate lymphoid cells as drivers of lung fibrosis in severe asthma

**DOI:** 10.3389/fimmu.2026.1957593

**Published:** 2026-08-25

**Authors:** Masaya Matsuda, Yuya Sannomiya, Osamu Kaminuma, Takeshi Nabe

**Affiliations:** 1 Laboratory of Immunopharmacology, Faculty of Pharmaceutical Sciences, Setsunan University, Osaka, Japan; 2 Department of Disease Model, Research Institute of Radiation Biology and Medicine, Hiroshima University, Hiroshima, Japan

**Keywords:** allergy, asthma, fibrosis, group 2 innate lymphoid cell, steroid resistance

## Abstract

Severe asthma is characterized by persistent airway inflammation, irreversible airway remodeling, and reduced responsiveness to glucocorticoids. Subepithelial fibrosis, a major determinant of progressive lung function decline, remains mechanistically undefined. Evidence identifies group 2 innate lymphoid cells (ILC2s) as key contributors to fibrotic airway remodeling. Importantly, ILC2s in severe asthma are not simply increased in number but functionally reprogrammed into a pathogenic, profibrotic state. We summarize evidence for pathogenic ILC2s and discuss three defining properties distinguishing them from homeostatic ILC2s. First, pathogenic ILC2s acquire a fibrogenic phenotype marked by increased IL-13, IL-4, IL-5, and amphiregulin, promoting fibroblast activation and matrix deposition. Second, they exhibit enhanced proliferative capacity driven by cell cycle-related (CDK4/6) and transcriptional (CDK8/19) cyclin-dependent kinases, expanding the pathogenic pool. Third, JAK–STAT5–Bcl-xL and PI3K–Akt–mTORC1 signaling confers resistance to glucocorticoid-induced apoptosis, enabling persistence despite corticosteroid therapy. Together, these properties provide a mechanistic framework linking chronic type 2 inflammation to persistent airway fibrosis. We also discuss therapeutic strategies targeting alarmins, cytokines, CDKs, and steroid-resistance signaling, and highlight open questions on ILC2 heterogeneity. Future single-cell and spatial transcriptomic studies will determine whether these properties arise within a common ILC2 population or distinct subsets, informing precision therapies for severe asthma.

## 1 Introduction

Asthma is a chronic inflammatory disease of the airways that affects approximately 300 million people worldwide (1). In most patients, asthma causes recurrent wheezing, dyspnea, and cough that are largely reversible. Symptoms are controlled by inhaled glucocorticoids combined with long-acting β2-adrenergic agonists (2). In 5% to 10% of patients, however, the disease remains refractory to this standard therapy (3), and these patients account for a disproportionate share of asthma-related healthcare costs (4). Severe asthma is not a single entity: while many patients exhibit type 2-high inflammation with airway eosinophilia that persists despite intensive glucocorticoid treatment, others show mixed eosinophilic-neutrophilic or type 2-low neutrophilic inflammation (5). In all of these settings, persistent airway inflammation drives structural change in the airway wall, a process known as airway remodeling, which comprises goblet cell metaplasia, thickening of the airway smooth muscle layer, and subepithelial fibrosis (6). The development of airway remodeling in severe asthma is still considered irreversible (7). Therefore, understanding the mechanisms that drive it is crucial for the prevention and treatment of severe asthma.

Type 2 inflammation is orchestrated primarily by type 2 helper T (Th2) cells and group 2 innate lymphoid cells (ILC2s) (8). Following activation by antigen-presenting cells, Th2 cells produce the type 2 cytokines interleukin (IL)-4, IL-5, and IL-13 (8). In contrast, ILC2s lack antigen-specific receptors but rapidly respond to epithelial cell-derived alarmins, including IL-33, IL-25, and thymic stromal lymphopoietin (TSLP), producing large amounts of type 2 cytokines (8). Although these cells contribute to host defense and tissue repair under physiological conditions (9), chronic inflammatory stimulation promotes the emergence of pathogenic Th2 cells and pathogenic ILC2s, which drive persistent tissue remodeling and disease progression. Morimoto et al. (10) demonstrated that amphiregulin-producing pathogenic memory Th2 cells promote airway fibrosis by inducing osteopontin production from eosinophils. More recently, Khan et al. (11) summarized the molecular mechanisms underlying pathogenic Th2 cell differentiation and provided a broader framework for understanding the emergence of pathogenic type 2 immune responses in allergic airway disease. Both pathogenic Th2 cells (10, 11) and ILC2s (12, 13) have been implicated in the development of airway remodeling in severe asthma, and accumulating evidence suggests that these cell populations cooperate to promote airway remodeling. While the pathogenic contribution of ILC2s is best established in the eosinophilic endotype, ILC2s are not phenotypically fixed: in the presence of IL-1β and IL-23 they can acquire an ILC3-like, IL-17-producing program (14), placing them within neutrophil-associated inflammatory circuits as well. The relevance of ILC2s to fibrotic remodeling is therefore likely to be context-dependent.

In this review, we focus on the emerging role of pathogenic ILC2s in fibrotic airway remodeling in the type 2-high setting, from which the mechanisms discussed below are predominantly drawn. We describe three characteristic features of pathogenic ILC2s that are thought to contribute to subepithelial fibrosis: their potent production of profibrotic mediators, enhanced proliferative capacity, and resistance to glucocorticoid-induced apoptosis, which underlies steroid resistance. By integrating recent findings from our group and others, we discuss how these pathogenic properties drive fibrotic remodeling and highlight their potential as therapeutic targets for severe asthma.

## 2 ILC2s as drivers of lung fibrosis in severe asthma

Under homeostatic conditions, lung ILC2s contribute to the maintenance of barrier integrity and tissue repair. IL-33 released from injured epithelium can activate ILC2s and induce amphiregulin-dependent tissue protection, while adventitial stromal cells help define tissue niches that support ILC2 survival and activation (15, 16). IL-13 supports airway epithelial repair and repair-associated epithelial turnover through IL-13 receptor (IL-13R) signaling (17), whereas amphiregulin promotes tissue protection and remodeling through epidermal growth factor receptor (EGFR) on epithelial and mesenchymal cells after injury (15). The capacity of ILC2s to activate fibroblasts is therefore a physiological component of wound healing that subsides once the epithelial barrier has been restored.

This homeostatic balance is disrupted in severe asthma. Smith et al. (18) reported that not only the total number of ILC2s but also the proportion of activated, IL-5^+^ IL-13^+^ ILC2s was significantly increased in the blood and airways of patients with severe asthma compared with those with mild disease, indicating that ILC2s undergo both quantitative expansion and qualitative activation as disease severity progresses. However, whether this shift directly contributes to fibrosis remains to be fully elucidated. To address this question, we recently developed a murine model that reproduces both mild and severe asthmatic phenotypes (12, 19).

In our established model, sensitized BALB/c mice are challenged intratracheally with ovalbumin (OVA) four times, and the antigen dose determines the outcome (12, 19). A low dose of 5 µg produces mucus accumulation, epithelial thickening, and airway hyperresponsiveness (AHR), all of which are significantly suppressed by daily dexamethasone treatment at a dose of 1 mg/kg. In contrast, a high dose of 500 µg produces marked airway remodeling that additionally includes subepithelial fibrosis, together with persistent AHR, and neither the airway remodeling nor the AHR is suppressed by the same dexamethasone regimen. Importantly, the number of lung ILC2s increases to a comparable extent in both models following antigen challenge; however, this increase is significantly suppressed by dexamethasone in the mild model, whereas no significant suppression is observed in the severe model (12). Moreover, ILC2s isolated from the severe asthma model produce approximately three-fold greater amounts of IL-5 and IL-13 than those from the mild asthma model (12). These findings indicate that, in the severe model, lung ILC2s acquire a pathogenic phenotype characterized by enhanced type 2 cytokine production and resistance to glucocorticoid-induced suppression. These cells are hereafter referred to as pathogenic ILC2s.

**Figure 1:**
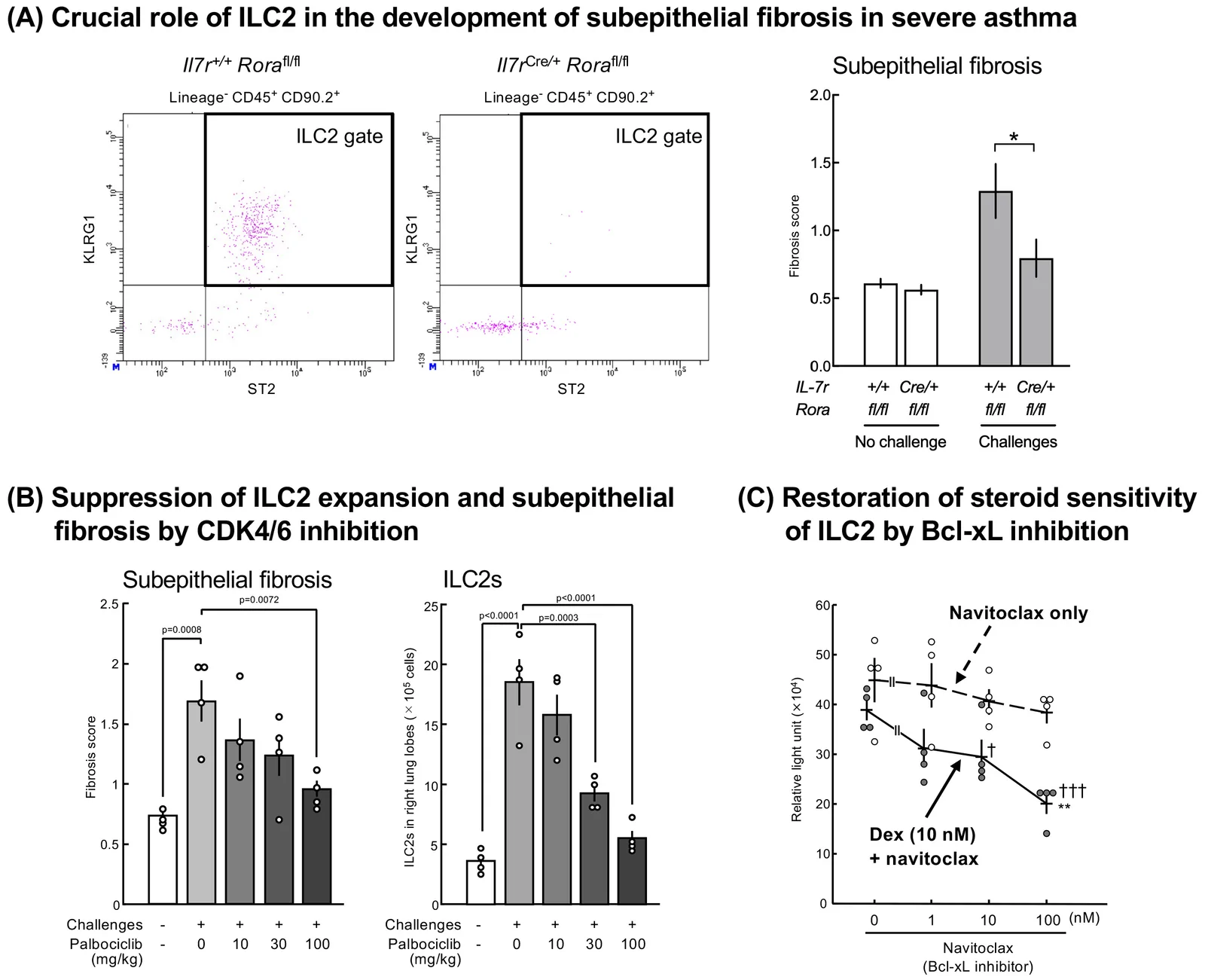
Pathogenic ILC2s drive subepithelial fibrosis in a murine model of severe asthma. **(A)** ILC2-deficient (*Il7r*^Cre/+^ *Rora*^fl/fl^) and control (*Il7r*^+/+^ *Rora*^fl/fl^) mice were sensitized and challenged intratracheally with ovalbumin (OVA). Lung ILC2s were analyzed by flow cytometry, and subepithelial fibrosis was scored on Masson trichrome-stained sections. Data are presented as mean ± SE (n = 4). *p < 0.05. **(B)** Sensitized BALB/c mice were challenged intratracheally with OVA and orally given palbociclib (10–100 mg/kg). **(C)** Lung ILC2s were cultured with IL-33, TSLP, and IL-7 in the presence of navitoclax (1–100 nM) with or without dexamethasone (Dex, 10 nM). **p < 0.01 versus the respective control (0 nM navitoclax); † and †††, p < 0.05 and 0.001 versus “navitoclax only”, respectively. Reproduced with permission from Matsuda et al., *J Immunol*, 2025 **(A)** (24), Matsuda et al., *J Pharmacol Exp Ther.*, 2025 **(B)** (41), and Shimora et al., *Immunology*, 2024 **(C)** (45).

To directly test whether ILC2s are required for the development of subepithelial fibrosis in severe asthma, we applied our severe asthma protocol to ILC2-deficient (*Il7r*^Cre/+^ *Rora*^fl/fl^) mice (20, 21) (**Figure 1A**), sensitizing and challenging them with the same high-dose antigen regimen used to induce severe asthma in wild-type mice. This model induces *Rora* deletion in *Il7r*-expressing lymphoid cells, and because RORα is an essential transcription factor for ILC2 development and maintenance (22, 23), it results in marked depletion of ILC2s. In these ILC2-deficient mice, subepithelial fibrosis failed to develop despite repeated high-dose antigen challenge (**Figure 1A**), even though the numbers of Th2 cells and neutrophils remained unchanged, indicating that this effect was specific to the loss of ILC2s rather than a broader impairment of the type 2 immune response (24). Otaki et al. (25) similarly established a spontaneous pulmonary fibrosis model using *Ifngr1*^-/-^ *Rag2*^-/-^ mice and demonstrated that fibrosis was completely abolished in ILC-deficient and IL-33-deficient derivatives of this strain. They further showed, using cocultures of wild-type-derived ILC2s and fibroblasts, that IL-33-activated ILC2s directly stimulate collagen production by fibroblasts. Hams et al. (26) demonstrated in both *S. mansoni* egg-induced and bleomycin-induced models that IL-25 signaling promotes pulmonary fibrosis. Using the S. mansoni model, they further established that ILC2-derived IL-13 is the critical effector driving collagen deposition. In a bleomycin-induced pulmonary fibrosis model, mice deficient in ST2, the receptor for IL-33, were protected from lung fibrosis, demonstrating that IL-33 promotes fibrotic remodeling by expanding ILC2s and alternatively activated macrophages (27).

Collectively, these findings support the concept that pathogenic ILC2s, rather than the simple expansion of the ILC2 population, are key drivers of fibrosis in severe asthma. Three defining properties distinguish pathogenic ILC2s from their homeostatic counterparts: enhanced profibrotic activity, excessive proliferative capacity, and resistance to glucocorticoid-induced apoptosis, each of which contributes to fibrotic airway remodeling.

## 3 Phenotypes of pathogenic ILC2s

### 3.1 Acquisition of a highly fibrogenic phenotype

ILC2s in severe asthma are not merely expanded in number but also qualitatively activated, producing greater amounts of IL-5 and IL-13. Our own studies further extend these observations. Lung ILC2s from the severe asthma model produced significantly more IL-13, one of the principal profibrotic mediators, than those from the mild asthma model (12). Notably, these ILC2s also acquired the capacity to produce IL-4, a cytokine that ILC2s express only marginally under conventional type 2 conditions. While IL-4 is classically regarded as an orchestrator of inflammation, primarily driving Th2 differentiation, IgE class switching, and eosinophil recruitment, it also contributes to airway remodeling and fibrosis through both direct and indirect mechanisms, including amplification of IL-13-associated responses, induction of procollagen, fibronectin, and tenascin in fibroblasts, promotion of myofibroblast differentiation in some contexts, and potentiation of TGF-β-linked matrix production (28). This acquired IL-4-producing capacity may therefore represent an additional, previously underappreciated contributor to ILC2-driven fibrosis. Rather than driving fibrosis on its own, IL-4 likely acts by boosting IL-13’s more direct fibrotic effects.

Pathogenic ILC2s directly promote fibroblast activation through the production of profibrotic mediators, including IL-13 and amphiregulin. IL-13 stimulates fibroblast differentiation toward a myofibroblast phenotype and enhances extracellular matrix production (29), whereas amphiregulin contributes to tissue remodeling through EGFR-dependent activation of mesenchymal cells (30). In addition to these direct effects, pathogenic ILC2-derived mediators promote fibrotic remodeling through interactions with alternatively activated macrophages. IL-13 and IL-4 produced by ILC2s promote M2 macrophage polarization (31, 32), generating alternatively activated macrophages that contribute to tissue remodeling through the production of profibrotic mediators, including TGF-β (33). These macrophage-derived signals further activate fibroblasts and promote extracellular matrix deposition (34). Consistently, IL-33-driven lung fibrosis has been shown to involve both ILC2 expansion and M2 macrophage polarization (27). Among these profibrotic mediators, TGF-β represents a central driver of fibroblast activation and extracellular matrix deposition (35). ILC2-derived IL-13 and IL-4 may therefore promote fibrosis not only through direct effects on fibroblasts but also by establishing a macrophage-mediated TGF-β-rich microenvironment, in which alternatively activated macrophages enhance fibroblast-to-myofibroblast transition and matrix production. Conversely, macrophages may also regulate pathogenic ILC2 responses. A recent review highlighted a distinct Ly6G^+^ Nur77^+^ lung macrophage population that senses allergens through protease-activated receptor 2 and produces cysteinyl leukotrienes (36), which can promote production of IL-4, IL-5 and IL-13 from ILC2s (37). Thus, pathogenic ILC2s and macrophages may form a reciprocal interaction network that amplifies type 2 inflammation and fibrotic remodeling.

Of note, ILC2s are also characterized by markedly increased IL-5 production (12). Neutralization of IL-5 alone with an anti-IL-5 antibody did not suppress the development of fibrosis, nor did dexamethasone alone; however, the combination of the anti-IL-5 antibody with dexamethasone significantly suppressed it (12). One plausible explanation for the limited effect of anti-IL-5 monotherapy is that, even after eosinophils are depleted, the ILC2s themselves persist and continue to produce IL-13 and IL-4, thereby sustaining the fibrotic program independently of the eosinophil-TGF-β route.

### 3.2 Acquisition of a high proliferative capacity: cell-cycle and transcriptional cyclin-dependent kinases

The second property is a heightened capacity to proliferate, which amplifies fibrogenic output by enlarging the effector pool. Malik et al. (38) reported that ILC2s derived from patients with severe asthma exhibit a high proliferative capacity. Moreover, Baba et al. (39) similarly demonstrated that cell growth-related genes are significantly upregulated in ILC2s derived from patients with severe asthma.

CDKs, which are serine/threonine kinases, are broadly classified into two subfamilies: cell cycle-related CDKs, which drive the G1-to-S phase transition, and transcriptional CDKs, which regulate RNA polymerase II activity (40). We have recently identified both classes of CDK as critical drivers of the proliferative and pathogenic phenotype of ILC2s in severe asthma (24, 41).

Focusing on the cell cycle-related subfamily, we demonstrated that CDK4 and CDK6 (CDK4/6) were markedly upregulated in lung ILC2s in a murine model of severe asthma (41). TSLP stimulation further enhanced CDK4/6 protein expression in ILC2s, driving their proliferation (41). *In vitro*, the CDK4/6 inhibitor palbociclib suppressed TSLP-induced ILC2 proliferation. *In vivo*, palbociclib attenuated the development of subepithelial fibrosis and suppressed ILC2 expansion in the lungs of the severe asthma model (41) (**Figure 1B**). These findings revealed that the TSLP receptor (TSLPR)–CDK4/6 axis is a novel pathway underlying ILC2-mediated fibrosis in severe asthma.

Regarding the transcriptional subfamily, we further showed that CDK8 and its paralog CDK19 (CDK8/19) contribute to ILC2 activation by phosphorylating the serine residues of signal transducer and activator of transcription 5 (STAT5), a modification distinct from the tyrosine phosphorylation mediated by Janus kinases (JAKs). Combined stimulation with IL-33 and IL-2 synergistically upregulated CDK8/19 expression in mouse lung ILC2s (24). *In vitro*, the CDK8/19 inhibitor AS3334366 suppressed IL-33/IL-2-induced ILC2 proliferation, production of the profibrotic mediators IL-13 and amphiregulin, and STAT5 serine phosphorylation in a concentration-dependent manner (24). *In vivo*, AS3334366 significantly ameliorated the development of lung fibrosis and suppressed ILC2 accumulation in OVA-challenged mice (24). This study revealed that CDK8/19 drives ILC2-mediated lung fibrosis via STAT5 serine phosphorylation, distinct from the CDK4/6-mediated proliferative pathway.

Thus, ILC2s exploit both arms of the CDK family to sustain their proliferative and profibrotic phenotype, positioning CDK inhibition as a therapeutic strategy.

### 3.3 Acquisition of steroid resistance through upregulation of an anti-apoptotic factor

The third property is enhanced survival, which turns a transient effector response into a persistent one. We recently reviewed this steroid-resistant phenotype of ILC2s in severe asthma in Immunology Letters (42), summarizing the signaling pathways underlying glucocorticoid unresponsiveness in both mouse and human ILC2s.

In humans, airway ILC2s from patients with severe asthma are refractory to glucocorticoid-mediated suppression, with activation of the TSLP–STAT5–Bcl-xL pathway identified as a key underlying mechanism (43). Consistently, a distinct CD45RO^+^ inflammatory ILC2 subset that is steroid-resistant and enriched in patients with severe asthma has also been reported (44), underscoring that glucocorticoid resistance is a defining feature of pathogenic ILC2s in human severe asthma.

Building on these human observations, our own studies using a murine model of severe asthma showed that the number of lung ILC2s was not reduced by dexamethasone treatment (12), indicating that these cells had acquired steroid resistance *in vivo*. Characterization of these ILC2s revealed marked upregulation of ST2 (the IL-33 receptor) and TSLPR at the protein level, together with significantly higher *Jak3* and *Stat5a* gene expression (12), suggesting that the IL-33 and TSLP signaling pathways were central to the acquisition of this steroid-resistant phenotype. Consistent with this, *in vitro* stimulation of ILC2s with IL-33, TSLP, or IL-7 alone showed that dexamethasone could still suppress ILC2 proliferation; however, this suppression was largely lost when IL-33 was combined with TSLP or IL-7, and disappeared almost completely in the presence of all three cytokines together, demonstrating that convergent signaling through these pathways drives steroid resistance (45).

Indeed, the importance of this pathway was pioneered by Kabata et al. (46), who demonstrated that the TSLP–STAT5–Bcl-xL pathway confers steroid resistance on mouse lung ILC2s, and that pharmacological inhibition of STAT5 with pimozide restores dexamethasone sensitivity. Building on this pathway, we intervened further upstream by targeting JAK, the kinase directly responsible for STAT5 activation, and obtained consistent results: A pan-JAK inhibitor reduced STAT5 phosphorylation and Bcl-xL expression and restored dexamethasone sensitivity *in vitro*. Navitoclax, a Bcl-xL inhibitor, likewise restored the sensitivity of ILC2 proliferation to dexamethasone in a concentration-dependent manner (**Figure 1C**). *In vivo*, both inhibitors suppressed the development of lung fibrosis when combined with dexamethasone (45).

We further showed that a parallel pathway acting downstream of IL-33, namely phosphoinositide 3-kinase (PI3K)–Akt–mechanistic target of rapamycin complex 1 (mTORC1), cooperates with JAK–STAT5 signaling to reinforce this steroid-resistant state. This axis not only contributes to Bcl-xL induction but also directly phosphorylates the glucocorticoid receptor (GR) at Ser234, a modification that impairs GR function and thereby blunts the responsiveness to dexamethasone (47). Together, JAK–STAT5–Bcl-xL and PI3K–Akt–mTORC1–GR pathways act cooperatively to establish a profound state of steroid resistance in ILC2s. In line with this central role of mTORC1 in ILC2 biology, Wang et al. (13) independently demonstrated that mTORC1 activation in lung ILC2s drives their pathogenic activation in a mouse model of allergic asthma, in that case by upregulating neuromedin U receptor 1 (NMUR1) and thereby sensitizing ILC2s to neuronally derived neuromedin U, further supporting mTORC1 as a convergent hub linking diverse environmental and immunological signals to ILC2 effector function.

Importantly, pathogenic ILC2s are unlikely to represent a uniform population but rather encompass functionally distinct subsets with different contributions to disease progression. Human studies have identified CD45RO^+^ inflammatory ILC2s (iILC2s), which exhibit enhanced activation, pathogenic cytokine production, and resistance to glucocorticoids compared with conventional ILC2s (44). These characteristics closely correspond to the pathogenic properties discussed in this review, particularly enhanced fibrogenic activity and steroid resistance. In addition, recent evidence indicates that ILC2s exhibit substantial plasticity and can acquire an ILC3-like phenotype characterized by IL-17A production under inflammatory conditions such as IL-1β and IL-23 stimulation (14). This ILC2-to-ILC3 transdifferentiation suggests that pathogenic ILC2 programs may extend beyond classical type 2 inflammation and contribute to mixed inflammatory endotypes of severe asthma. Whether the fibrogenic, proliferative, and steroid-resistant properties described here are confined to specific ILC2 subsets or represent acquired states shared across multiple populations remains an important question. Defining the relationship between ILC2 heterogeneity and pathogenic programs will be essential for developing precision therapies targeting severe asthma.

**Figure 2:**
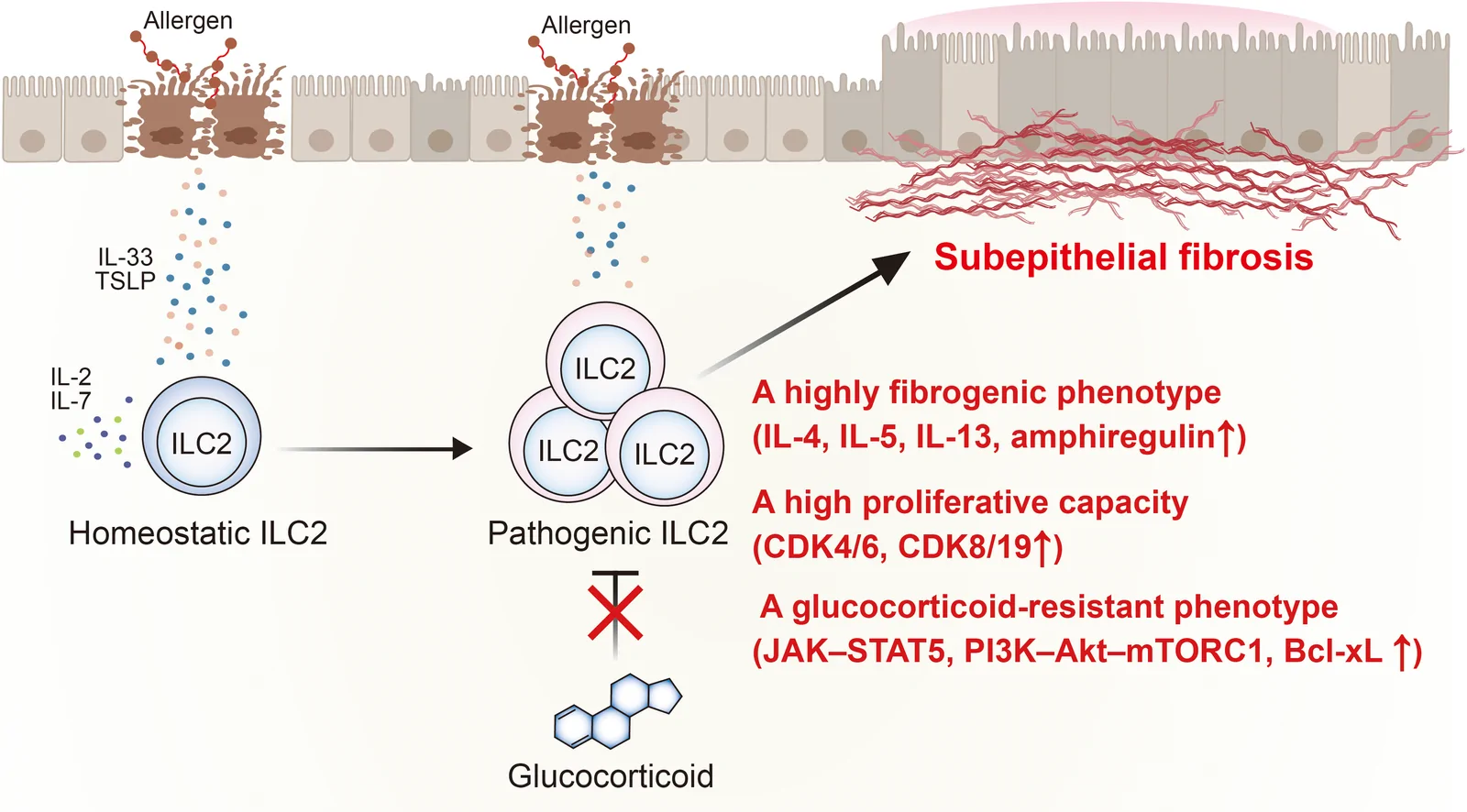
Proposed model illustrating the acquisition of pathogenic ILC2 phenotypes in severe asthma. Under homeostatic conditions, lung ILC2s contribute to tissue repair and maintenance of epithelial integrity following activation by epithelial-derived cytokines. In severe asthma, persistent exposure to allergens induces the production of IL-33, TSLP, IL-2, and IL-7, which promote the transition of homeostatic ILC2s into pathogenic ILC2s. These cells acquire three characteristic pathogenic properties: (i) a highly fibrogenic phenotype characterized by increased production of IL-4, IL-5, IL-13, and amphiregulin; (ii) enhanced proliferative capacity mediated by CDK4/6 and CDK8/19; and (iii) glucocorticoid resistance associated with activation of the JAK–STAT5–Bcl-xL and PI3K–Akt–mTORC1 signaling pathways. Collectively, these pathogenic properties promote subepithelial fibrosis and persistent airway remodeling in severe asthma.

Collectively, these three properties are not independent phenomena but integrated components of a single pathogenic program (**Figure 2**). The fibrogenic secretory shift supplies the mediators that drive tissue remodeling; the proliferative expansion, governed by both cell cycle-related and transcriptional CDKs, enlarges the pool of cells producing these mediators; and the acquisition of steroid resistance, through the JAK–STAT5–Bcl-xL and PI3K–Akt–mTORC1–GR axes, ensures that this fibrogenic, proliferative population persists despite glucocorticoid therapy. Although these pathogenic properties have been mainly characterized in murine models, accumulating evidence from human studies indicates that similar features contribute to severe asthma pathogenesis. The current evidence supporting each pathogenic property of ILC2s in mouse models and patients with severe asthma is comparatively summarized in **Table 1**.

**Table 1:** Comparative evidence supporting pathogenic properties of ILC2s between mouse models and patients with severe asthma.

Pathogenic property	Associated molecules/pathways	References	
		Mouse	Human
Fibrogenic phenotype	IL-4, IL-5, IL-13, amphiregulin	(12)	(18, 39)
Proliferative capacity	CDK4/6	(41)	–
	CDK8/19	(24)	–
Glucocorticoid resistance	JAK–STAT5–Bcl-xL	(45, 46)	(43, 44)
	PI3K–Akt–mTORC1	(47)	–

## 4 Conclusion and future perspectives

Several important questions remain unresolved regarding the biology and therapeutic targeting of pathogenic ILC2s in severe asthma. Although accumulating evidence indicates that pathogenic ILC2s contribute to airway fibrosis and glucocorticoid resistance, it remains unclear whether their fibrogenic, proliferative, and steroid-resistant properties represent independent functional states or are co-acquired within a common pathogenic program. Resolving this issue will require high-resolution approaches such as single-cell RNA sequencing, spatial transcriptomics, and lineage-tracing analyses to define the cellular and functional heterogeneity of ILC2s in severe asthma.

These mechanistic insights also highlight several potential therapeutic opportunities targeting different aspects of pathogenic ILC2 biology. Current biologic therapies for severe asthma, including dupilumab (IL-4Rα blockade), mepolizumab (IL-5 blockade), and tezepelumab (TSLP blockade), primarily target upstream cytokine pathways that initiate or amplify type 2 inflammation. Although these therapies have substantially improved disease control in many patients, they may not fully address established pathogenic ILC2 states characterized by enhanced proliferation, fibrogenic activity, and glucocorticoid resistance. In contrast, targeting CDK-dependent proliferative signaling or intracellular survival pathways such as JAK–STAT5–Bcl-xL and PI3K–Akt–mTORC1 may provide complementary approaches by directly modulating the cellular programs that maintain pathogenic ILC2 functions. However, these strategies remain largely at the preclinical stage for asthma, and their therapeutic potential, safety, and applicability in combination with existing biologics require further investigation. Future studies identifying molecular signatures associated with fibrogenic, proliferative, or steroid-resistant ILC2 phenotypes may therefore facilitate a more precise and mechanism-based approach to treatment selection.

Several important challenges remain before these concepts can be translated into clinical practice. First, much of the current mechanistic evidence linking CDK4/6, CDK8/19, JAK–STAT5–Bcl-xL, and PI3K–Akt–mTORC1 signaling to pathogenic ILC2 function has been generated in murine models, and confirmation in human ILC2s is still required. Second, it remains unknown whether pharmacological targeting of these pathways will provide additive or synergistic benefits when combined with existing therapies, including glucocorticoids and biologics. Third, the relative contribution of individual pathogenic properties to clinically relevant outcomes, such as exacerbation frequency, lung function decline, airway remodeling, and corticosteroid dependence, has not yet been defined. Finally, reliable biomarkers capable of identifying the dominant pathogenic ILC2 program in individual patients are lacking but will be essential for implementing precision medicine approaches based on disease mechanisms rather than clinical phenotype alone.

Taken together, recent advances suggest that pathogenic ILC2s are not simply expanded effector cells but are functionally reprogrammed populations that integrate multiple disease-promoting properties. Defining how these pathogenic programs are organized at the single-cell level, how they evolve during disease progression, and how they differ among asthma endotypes will be critical for the development of next-generation therapeutic strategies. Continued integration of single-cell technologies, spatial profiling, and functional validation in human tissues is expected to refine our understanding of pathogenic ILC2 biology and ultimately enable more precise interventions for patients with severe, steroid-resistant asthma.
